# Improvement of Gel Properties of *Nemipterus virgatus* Myofibrillar Protein Emulsion Gels by Curdlan: Development and Application to Emulsified Surimi

**DOI:** 10.3390/gels11090753

**Published:** 2025-09-17

**Authors:** Zhiqin Wu, Yongyan Qu, Ouhongyi Li, Soottawat Benjakul, Aimei Zhou

**Affiliations:** 1 College of Food Science, South China Agricultural University, Guangzhou 510642, China; wuzhiqin1983@163.com (Z.W.); quyongyan0919@163.com (Y.Q.); 13626909168@163.com (O.L.); 2 Department of Food Technology, Faculty of Agro-Industry, Prince of Songkla University, Hat Yai, Songkhla 90112, Thailand; soottawat.b@psu.ac.th

**Keywords:** emulsion gel, composite network, textural properties, surimi

## Abstract

This study aims to improve the gel properties of *Nemipterus virgatus* myofibrillar protein (MP) emulsion gels by Curdlan (Cur) and investigate the effect of the emulsion gels on the quality of emulsified surimi gels. The effects of different concentrations of Cur on the gel properties of MP emulsion gels were investigated. Fourier transform infrared (FTIR) results indicated that intermolecular interactions between Cur and MP were primarily hydrogen bonds. Cur enhanced the adsorption capacity of MP at the oil/water interface, inducing the formation of a more uniform and dense composite network structure in Cur/MP emulsion gels. Adding 6% (*w*/*v*) of Cur significantly increased the hardness, gel strength, water-holding capacity (WHC) and rheological properties of the gel. In addition, microstructural images showed that MP formed a complex interpenetrating network with Cur, thus enhancing the gel network skeleton. Low-field NMR confirmed that the addition of Cur decreased water mobility in the emulsion gel system. Compared to the direct addition of oil, the application of Cur/MP emulsion gels to surimi significantly improved the texture, gel strength, and WHC of the surimi gel. These findings provide a reference for the development of myofibrillar protein emulsion gels and broaden their potential application in the food industry.

## 1. Introduction

Dietary fat is an important nutrient for maintaining human physiological functions and provides energy for human metabolism. Saturated fatty acids are the most common type of lipid in daily consumed meat products, but excessive intake may induce cardiovascular diseases, coronary heart disease, type 2 diabetes, and obesity [1]. Replacing saturated fats with plant oils or marine liquid oils in surimi products confers health benefits [2]. However, their liquid state results in a softer surimi texture. Furthermore, direct oil addition hinders protein cross-linking, thereby disrupting the surimi gel network structure [3]. Therefore, developing healthy and sustainable fat substitutes with fat-mimetic textures has become urgent to meet growing consumer demand for natural and health-promoting alternatives [4]. In recent years, emulsion gels have been widely recognized as a promising fat substitute due to their unique structural and functional properties [5]. Emulsion gels possess dual emulsion and gel characteristics, which reduce oil droplet size while helping maintain fat-mimicking sensory properties [6]. Emulsion gels formulated with vegetable oils and natural materials (proteins, polysaccharides, or oligosaccharides) offer advantages including safety, health benefits, low calorie content, and richness in unsaturated fatty acids [7]. Among them, proteins are ideal components for the development of emulsion gels due to their dual capabilities, excellent ability to stabilize the water/oil interfaces, and gel-forming properties [8]. Animal proteins, especially fish proteins, have garnered increasing attention due to their high nutritional value and functional properties. As a high-quality protein resource, fish protein contains essential amino acids (lysine, etc.) and exhibits advantages such as easy digestibility and hypoallergenicity, making it suitable for diverse populations requiring nutritional supplementation [9]. However, its high-value applications in emulsification and delivery systems remain insufficient. Myofibrillar protein (MP), the primary structural protein of fish muscle, demonstrates excellent gel-forming ability and emulsification properties. MP not only forms intermolecular cross-linked networks, but also its abundant hydrophobic groups anchors on the surface of oil droplets to form interfacial protein films that prevent oil droplet aggregation [10]. However, pure MP stabilized emulsion gels are susceptible to thermal denaturation and aggregation of MP molecules during heating, leading to unsatisfactory product quality [11]. Additionally, the texture of pure MP emulsion exhibits insufficient texture firmness. Pei et al. [12] reported that while the addition of MP emulsion gels improved the water/oil retention of fish sausage, they reduced the gel strength of surimi gel, potentially diminishing consumer sensory satisfaction. Previous studies demonstrate that polysaccharides can enhance the functional properties of proteins by strengthening the protein gel network through covalent or non-covalent interactions [13]. Zhao et al. [5] demonstrated that the addition of *Konjac glucomannan* improved the gel properties of soybean isolate protein emulsion gels through physical bonding. In order to enhance the thermal stability, emulsification, and gel strength of MP emulsion gels, polysaccharide incorporation is a natural and safe strategy.

Curdlan (Cur), a linear β-1,3-glucan produced by microbial fermentation, which exhibits the advantages of food safety, water-holding capacity, thermal stability, and gel-forming ability [14]. Cur has been widely used to improve the gelation properties of protein gels by hydration and filling effects, including water-holding capacity and gel strength [15]. Cur is insoluble in cold water, but heating Cur aqueous suspension to 55–60 °C or above 80 °C yields thermo-reversible and thermo-irreversible gels, respectively. At temperatures above 80 °C, the disordered coil-like molecular conformation of Cur transforms into a stable and ordered triple-helix structure and undergoes hydration [16]. Upon cooling, this triple-helix molecular structure forms a thermally irreversible three-dimensional network through strong intermolecular hydrogen bonding interactions that encapsulate water in the network [17]. Cur after high temperature heating exhibits excellent gel strength and mechanical stiffness [18]. It had been shown that strategically mixing Cur with soybean isolate proteins yields a desirable texture comparable to animal fat, which prevented emulsion gels from forming an imbalanced texture (excessive rigidity or flexibility) [19]. Furthermore, unfolded Cur is able to enhance protein gel strength through hydrogen bonding and hydrophobic interactions with MP [20]. Although the effect of Cur on the properties of MP gels has been extensively studied, there is a lack of reports on emulsion gels prepared by mixing fish MP with Cur as the aqueous phase. Meanwhile, the effect of replacing unemulsified vegetable oils with MP/Cur emulsion gels on the gel properties of surimi products unknown. *Nemipterus virgatus* has become one of the major raw materials in surimi processing due to its high nutritional value, rapid growth rate, and high yield, ranking as the second most important resource for surimi production after Alaska pollock [21]. As an abundant and low-value marine fish species, the development and utilization of *Nemipterus virgatus* for high-value applications is of significant research importance. Therefore, this study used *Nemipterus virgatus* as the raw material for the extraction of MP and the preparation of surimi gel.

Accordingly, this study aimed to investigate the formation and gel properties of emulsion gels prepared by blending MP with Cur, as well as the effect of such emulsion gels on the gel characteristics of surimi products. Cur/MP emulsion gel was prepared using a one-step method and characterized for their gel properties such as structure, texture and water retention capacity. Subsequently, surimi gels were prepared by mixing Cur/MP emulsion gels containing different Cur contents with surimi, and their effects on the properties of surimi gels were evaluated compared to the direct addition of soybean oil. This study provides an efficient strategy for improving the physical properties of fish MP emulsion gels. The results are expected to provide theoretical references to advance the development of MP emulsion gels and their application in surimi products.

## 2. Results and Discussion

### 2.1. Characterization of Cur/MP Mixtures

#### 2.1.1. FTIR

**Figure 1:**
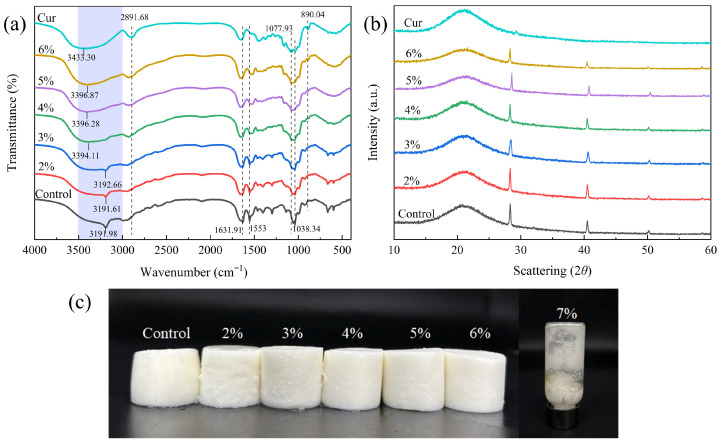
FTIR spectrum (**a**), XRD spectrum (**b**) appearance and (**c**) of Cur/MP emulsion gels prepared with different concentrations of Cur.

The intermolecular interactions of Cur/MP complexes were investigated by FTIR spectroscopy. As shown in Figure 1a, the characteristic bands of the samples at about 3000–3500 cm^−1^ corresponded to O–H or N–H stretching vibrations, while the absorption bands at 2990–2890 cm^−1^ were attributed to C–H stretching vibrations [22]. Typical protein bands were observed: the absorption band at 1680–1630 cm^−1^ corresponded to C–O stretching vibration (amide I band), the absorption band at 1560–1530 cm^−1^ corresponded to N–H vibration (amide II band), and the absorption band at 1450–1240 cm^−1^ corresponded to N-H deformation and C–H stretching vibration (amide III band) [19]. In addition, the absorption peak at 1038.34 cm^−1^ in the spectrum of MP represented C–O or C–C stretching vibration [23]. For Cur, the absorption peaks at 1077.93 cm^−1^ (C–O glycosidic bond) and 890.04 cm^−1^ (β-1, 3-D-glucan) were observed [24]. Notably, no new absorption peaks were observed in the FTIR spectra of Cur/MP complexes with different Cur concentrations compared to pure MP (Figure 1a). This suggested that no new functional groups were formed between Cur and MP molecules and that no covalent interactions occurred between the compounds. In the Cur/MP complexes profile, the signals of the unique absorption peaks of Cur (3433 cm^−1^, 2891.68 cm^−1^, 1077.93 cm^−1^ and 890.04 cm^−1^) were intensified and the signals of the MP absorption peaks (3191.98 cm^−1^, 2989.77 cm^−1^, 1631.91 cm^−1^, 1553 cm^−1^ and 1038.34 cm^−1^) were diminished as the concentration of Cur was increased. The absorption bands of Cur/MP complexes gradually changed from being similar to pure MP (Cur ≤ 3%) to being similar to pure Cur (Cur > 3%), indicating that Cur gradually became the dominant component of the complexes, and intermolecular interactions were enhanced with the increase in Cur concentration. Blue shifts in hydroxyl absorption peaks of the Cur/MP complexes were observed in comparison to the spectrum of Cur and red shifts compared to the spectrum of MP [24]. These wavelength shifts confirmed hydrogen bonding between Cur and MP. Similar findings were found by Zhu et al. [20] in a study of TGase and curdlan to improve the gel strength of surimi gels.

#### 2.1.2. XRD

The crystal structures of Cur/MP complexes with different Cur contents were characterized by XRD to gather information related to the interaction between Cur and MP (Figure 1b). Pure Cur exhibited a broad diffraction peak at about 2*θ* = 21° and a weak peak at 2*θ* = 29°, confirming the amorphous structure of Cur [25]. Pure MP displayed an amorphous band at 2*θ* = 21°. The presence of differential amorphous peaks in Cur and MP maps also indicated the inconsistent arrangement of atoms in proteins or polysaccharides [26]. Notably, the disappearance of the 2*θ* = 29° diffraction peaks in the profile of Cur-MP complexes compared to pure Cur might be caused by the interaction of Cur with MP disrupting the original crystal structure domains of Cur [24]. The profile of Cur/MP complexes was similar to that of pure MP and the signal intensity of all the diffraction peaks of Cur/MP polymer varied with the difference in the concentration of Cur, which suggested that Cur may have altered the crystalline properties of the complexes, and that Cur-MP interactions resulted in conformational changes in MP [27]. The intensity of the diffraction peaks of Cur/MP complexes with a Cur concentration of 2% tended to increase but tended to decrease slightly with continued increase in Cur concentration. This may be attributed to the interaction between excess Cur [28]. The strong interactions formed by high concentrations of Cur prevent spontaneous interactions between Cur-MP and MP molecules [28]. Collectively, the experimental results of XRD were in agreement with the existence of natural interactions in Cur/MP complexes as evidenced by FTIR spectroscopy. Furthermore, XRD analysis confirmed that the addition of Cur enhanced the intermolecular interactions within the emulsion gel system, thereby contributing to improved gel strength. This enhancement was further reflected in TPA (Table 1) and rheological properties.

### 2.2. Cur/MP Emulsion Gels Property Determination

#### 2.2.1. TPA and Gel Strength

The texture of the food affects the acceptance of consumer, and TPA can present a comprehensive picture of the texture condition of emulsion gels from a mechanical point of view. As shown in Figure 1c, with increasing Cur concentration, Cur/MP emulsion gels exhibited a harder solid morphology compared to the pure MP emulsion gel. In addition, the emulsification system collapsed when the Cur concentration reached 7% and subsequent experiments could not be performed, necessitating the maximum concentration of Cur was set at 6% in this study. Hardness is determined by the ability of the sample to resist external pressure, chewiness and springiness respond to the combined texture properties of the food when it is chewed [25]. Gumminess reflects the force of the sample to resist external separation, and cohesion responds to the strength of the internal interactions of the sample when it resists damage from external forces. As reflected in Table 1, the hardness, chewiness, springiness, gumminess and cohesiveness of Cur/MP emulsion gels were significantly increased (*p* < 0.05) with increasing Cur content. At the maximum concentration of Cur (6%), the chewiness, springiness, gumminess and cohesiveness of Cur/MP emulsion gel reached the maximum value and increased by 488.86%, 758.85%, 13.95%, 286.01% and 28.67%, respectively, over the pure MP emulsion gel. The results indicated that Cur could significantly improve the texture and mouthfeel of Cur/MP emulsion gels and enhance their mechanical properties (*p* < 0.05). The improvement in textural properties could be attributed to the strong hydrogen bonding interactions between Cur molecules in the continuous phase of the emulsion gel to form an elastic three-dimensional network induced at high temperatures, enhancing the strength of the MP gel network structure. This is consistent with the findings from Figure 1a FTIR, where the hydrogen bond signal peak gradually intensifies with increasing Cur concentration, indicating that Cur addition enhances the hydrogen bonding interactions between Cur and MP molecules. The heating treatment induced the gradual stretching of the Cur molecular conformation from the curled state, and the resulting unfolded Cur and MP molecules generated noncovalent interactions and formed a dense microstructure, which enhanced the textural properties of emulsion gels [29]. A large increase in the Cur content led to a significant increase in the intermolecular forces between Cur molecules and the formation of a Cur-dominated composite gel, and a high concentration of Cur reduced the interference of MP on the intermolecular interactions between Cur molecules, as evidenced by the FITR spectrum.

Gel strength is an important index to assess the performance of gels and reflects the density of the gel structure. The gel strength of all Cur/MP emulsion gels were significantly higher than that of the control (*p* < 0.05), with a positive correlation between gel strength and Cur concentration. The gel strength reached a maximum value of 734.89 g·mm at a Cur concentration of 6%, representing a 594.26% increase over pure MP emulsion gels. The textural properties and the enhancement of gel strength of Cur/MP emulsion gels indicated that the density of the gel structure was enhanced by Cur. The results were consistent with the TPA. The enhancement of textural properties and gel strength of Cur/MP emulsion gels suggests that Cur is able to improve the network structure of the gels. This is due to the thermogenic gel properties of Cur. Cur can form thermally irreversible highly solidifying gels and enhance the mechanical properties of emulsion gels through hydration and filling.

#### 2.2.2. Water-Holding Capacity (WHC)

**Figure 2:**
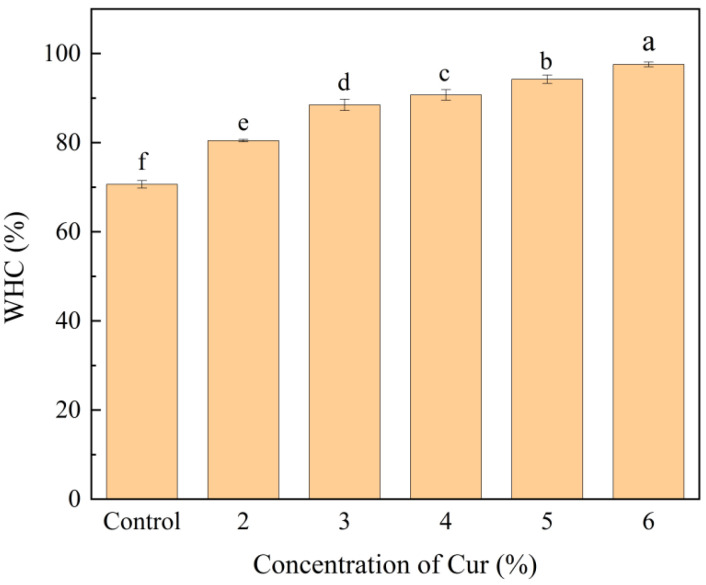
WHC of Cur/MP emulsion gels prepared with different concentrations of Cur. Different letters indicate significant differences (*p* < 0.05).

The water holding capacity of emulsion gels represents the ability of emulsion gels to immobilize and retain water. The ability of the gel network to retain water also indirectly reflects the state of the gel network structure [30]. As shown in Figure 2, the addition of Cur significantly increased the WHC of pure MP emulsion gels (*p* < 0.05). At 6% Cur, WHC reached a maximum value of 97.56%, representing a 38.11% increase compared to pure MP emulsion gel (70.64%). Cur retained water by exposing a large number of hydroxyl groups that combine with water molecules to form hydrogen bonds during gel formation [31]. The addition of Cur enhanced hydrogen bonding interactions with MP will improve the water binding capacity of emulsion gels [20]. In addition, Cur produced a three-dimensional network structure when forming gels. The results from Figure 1a FTIR and Figure 1b XRD collectively demonstrated that the addition of Cur enhanced the intermolecular interactions between Cur and MP within the emulsion gel, which was a key factor contributing to the improved gel network structure. Furthermore, such a dense and compact network played a critical role in enhancing the WHC of the gel [32]. As the concentration of Cur increases, the resultant denser and multi-hierarchical network structure from the interaction of Cur and MP contributed to generating stronger capillary forces to trap water.

#### 2.2.3. Rheological Behavior

**Figure 3:**
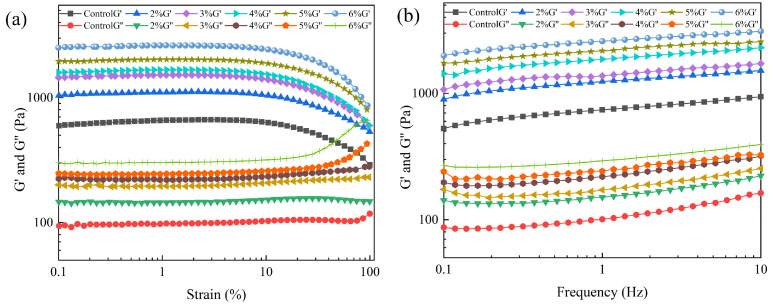
Strain scans (**a**) and frequency scans (**b**) of Cur/MP emulsion gels prepared with different concentrations of Cur.

Information on the dynamic rheological properties of emulsion gels at increasing strain at a fixed frequency was obtained by strain scanning. The energy storage modulus (G′) and loss modulus (G″) reflected the elastic and viscous properties of the samples, respectively. The results are shown in Figure 3a, where the energy storage modulus (G′) is higher than the loss modulus (G″) in the linear viscoelastic region (LVR) for all experimental groups, indicative of their elastic behavior at low strains. Both G′ and G″ of the emulsion gels progressively increased with Cur concentration. This trend indicated that Cur enhanced the solid-like characteristics of MP emulsion gels. It was observed that all samples exhibited relatively stable G′ values and G″ values in the 1% strain range, due to the structural integrity of the emulsion gel. Consequently, a strain of 1% was selected for subsequent frequency and temperature scans within the LVR.

The frequency scans of the emulsion gels were able to respond to the effect of the available gel on the viscoelasticity of the MP emulsion gel network structure. As shown in Figure 3b, the G′ values of all experimental groups were higher than the G″ values throughout the frequency range, which indicated that all samples exhibited elastic gel properties. The G′ values of the emulsion gels increased with increasing Cur concentration, peaking at a Cur addition of 6%. This trend was consistent with the strain scan results, indicating that Cur improved the gel strength of the MP emulsion gels. The enhancements of gel properties of gel systems were associated with the improvement of gel network structures. Cur formed a dense three-dimensional network with MP in the emulsion gel matrix, thus enhancing the viscoelastic behavior [33]. Cur molecules were linked to the protein network to enhance the stability of the emulsion gel network.

#### 2.2.4. LF-NMR Relaxation

**Figure 4:**
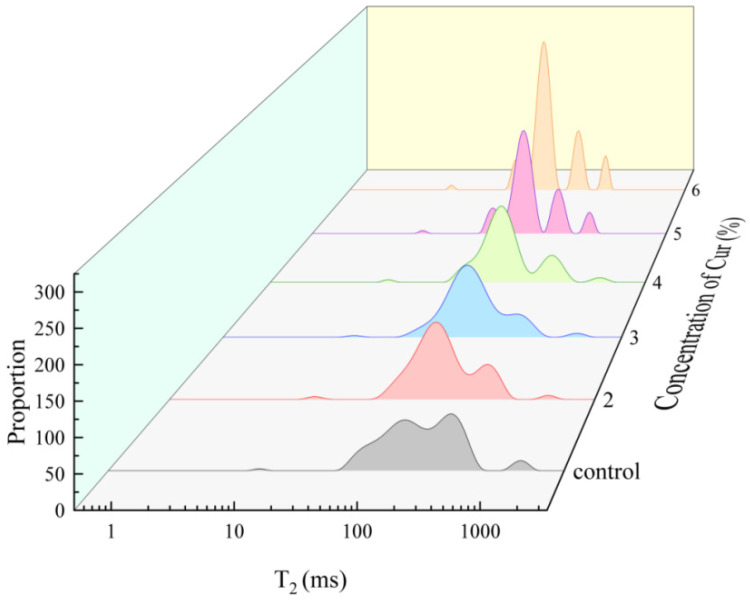
Spin-spin relaxation spectra (T_2_) of Cur/MP emulsion gels prepared with different concentrations of Cur.

**Figure 5:**
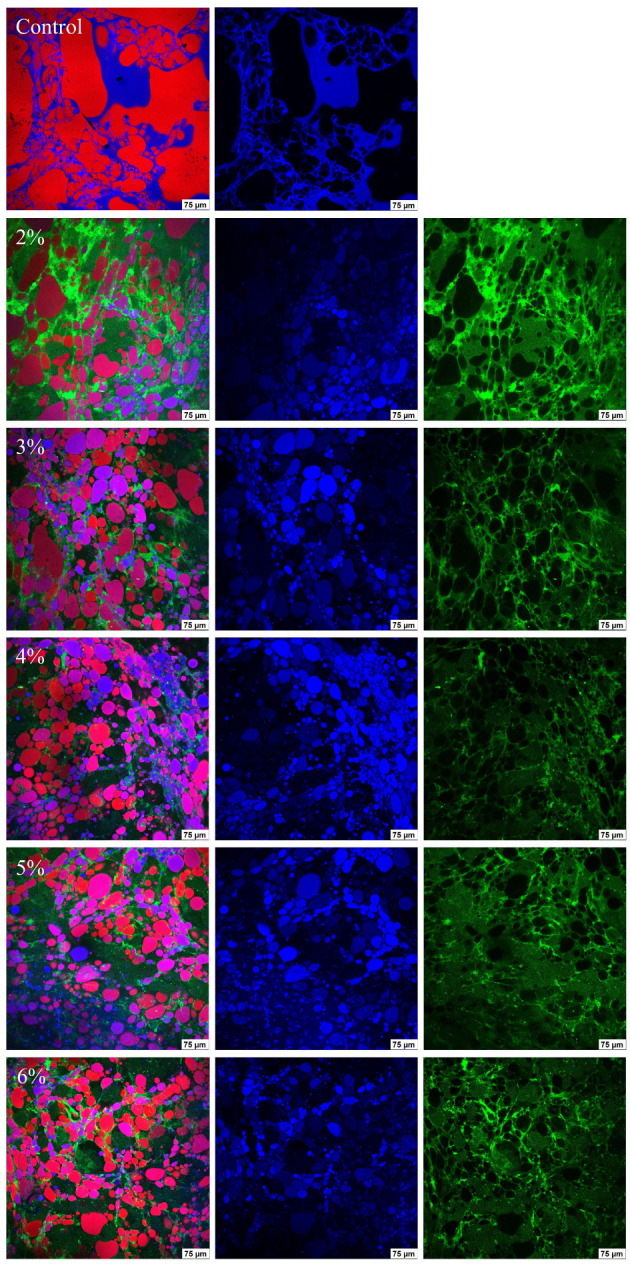
CLSM images of Cur/MP emulsion gels prepared with different concentrations of Cur.

**Figure 6:**
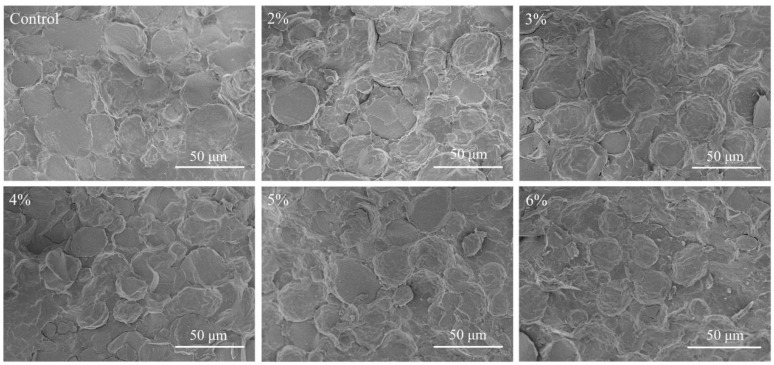
Cryo-SEM images of Cur/MP emulsion gels prepared with different concentrations of Cur.

Transverse relaxation time (T_2_) can characterize the binding of hydrogen protons in samples and thus reflects the strength of water mobility in the sample. The T_2_ of emulsion gels was determined by low field nuclear magnetic resonance (LF-NMR) to analyze the distribution and migration of water. A shorter relaxation time represents a smaller degree of freedom for the hydrogen proton. Figure 4 shows the relaxation peaks of three different hydrogen proton signals for emulsion gel samples: the T_21_ (0.5–12 ms) was associated with water/oil tightly bound by the macromolecules, the T_22_ (12–900 ms) corresponded to immobilized water/oil in the emulsion gel network, and the T_23_ (>900 ms) represented free water/oil [26]. With the increase in Cur content, the relaxation times of T_22_ and T_23_ of Cur/MP emulsion gels were gradually shortened compared to the control group. In particular, the relaxation time of peaks in the emulsion gel samples did not exceed 1000 ms at Cur concentrations ≥5%, which was closely related to the Cur induced unique stability of MP emulsion gels. The results suggested that the addition of Cur to the MP emulsion gel system decreased the degree of freedom of hydrogen protons and enhanced the binding capacity to water/oil. To further analyze the water distribution in each experimental group, the relative contents of water in different states are shown in Table 2. A_21_, A_22_ and A_23_ represented the relaxation peak relative areas of T_21_, T_22_ and T_23_, respectively. The A_21_ of all groups had no significant difference (*p* > 0.5) and was much lower than the other relaxation peaks (not more than 1% of the total content), indicating that the content of Cur had no significant effect on the bound water/oil of MP emulsion gels. The addition of Cur resulted in a significant increase in A_22_ along with a significant decrease in A_23_ compared to the control (*p* < 0.5). The higher the concentration of Cur the higher the proportion of immobilized water in the system, and no free water was detected at higher concentrations of Cur (5–6%). These changes indicated that the addition of Cur resulted in the migration of free water to immobilized water in MP emulsion gels, which was consistent with the results of WHC. This may be because the dense gel network induced by Cur was helpful to improve the water retention of MP emulsion gels, a finding further supported by CLSM (Figure 5) and Cryo-SEM (Figure 6) images. Combined with the microstructural imaging results, it was demonstrated that Cur/MP emulsion gels formed a denser network, which increased capillary forces and thereby enhanced the retention of both water and oil. The Cur molecule was rich in hydroxyl groups capable of binding more free water through hydrogen bonding interactions. In addition, the complex network system formed by the interpenetration of Cur and MP networks also reduced the water/oil mobility [20].

#### 2.2.5. CLSM

The influence of Cur on the morphological conformation of MP emulsion gels was observed by CLSM. As shown in Figure 5, Cur labeled green, MP labeled blue and oil droplets labeled red can be observed to determine the spatial distribution of these three substances. The image of pure MP emulsion gel showed that the particle size and distribution of oil droplets in the gel network structure were severely inhomogeneous, and there were some larger irregularly shaped oil droplets, which impeded the cross-linking between MP molecules and led to the creation of large voids in the microstructure of the pure MP emulsion gels, thus decreasing their ability to retain water and oil. With the addition of Cur (2–6%), Cur/MP emulsion gels achieved a more uniform microstructure, the shape of the oil droplets showed a more regular spherical shape, and the particle size of the oil droplets were smaller compared with that of the pure MP emulsion gel. In terms of the interfacial state, from CLSM images of Cur/MP emulsion gels, it could be observed that part of the blue fluorescence signals overlapped with the red color, and MP was adsorbed on the surface of the oil droplets to form an interfacial layer. Moreover, the oil droplet was surrounded by strong blue and green fluorescence, and part of the blue and green fluorescence signals overlapped with red to form purple and orange fluorescence, indicating that the interface around the oil droplets were totally encapsulated by Cur and MP. In addition to these, more Cur and MP filled in the voids among the oil droplets and connected with the Cur and MP at the droplet interface, which helped to form a spatial barrier to stabilize the droplets. Cur has been shown to have certain hydrophobic and emulsifying properties [34]. Incorporation of an appropriate amount of Cur in the continuous phase contributed to enhancing the tight adsorption of MP on the surface of oil droplets, intertwining with Cur at droplet-to-droplet interfaces. This interpenetrating network structure effectively restricted the movement of oil droplets, preventing aggregation and thereby improving the stability of the emulsion gel [35]. Specifically, molecular chains of Cur were interconnected with MP in the continuous phase of emulsion gels through hydrophobic interactions, hydrogen bonding and van der Waals forces to form a more stable three-dimensional network structure [19]. At the same time, Cur also formed aggregates to fill in the emulsion gel networks. The synergistic effect of bridging and flocculation structures contributed to the formation of a more stable emulsion system [20]. In summary, in addition to the adsorption of Cur and MP at the oil/water interfacial layer (MP predominantly), another part of Cur and MP filled the space between the oil droplets and acted as the emulsion gel skeleton, thus improving the stability of the emulsion gel.

#### 2.2.6. Cryo-SEM

Cryo-SEM was used to analyze the reasons for the changes in the gel properties of emulsion gels from a microscopic point of view. Figure 6 shows Cryo-SEM images of emulsion gels with different Cur contents. Some irregular, large and unevenly distributed oil droplets could be observed in the microstructure of pure MP emulsion gels, the protein interfacial film could not be uniformly and tightly wrapped around the surface of the oil droplets, and the structure of the gel network was relatively loose and disordered. This phenomenon may be due to the fact that the amount of MP was not sufficient to completely cover all the oil droplets formed during the homogenization process [36]. As the Cur concentration increased (2–6%), the oil droplet size was gradually reduced and tightly wrapped by the interfacial film. The Cur/MP emulsion gels showed a more homogeneous and compact network structure. The possible reasons affecting the emulsification effect of Cur/MP emulsion gels are as follows. On the one hand, Cur had a thickening effect on the emulsion gel system, which prevented oil droplets from agglomerating by strengthening the spatial site resistance during high-speed shear. It has been reported that the higher viscosity aqueous phase in the emulsified system contributes to the formation of smaller oil droplets [37]. On the other hand, Cur increased the network structure strength of Cur/MP emulsion gels by self-assembling from a mixture of single helices and loosely interconnected triple helices to form a more cohesive rod-like triple-helical structure during thermal treatment [6]. These would significantly alter the microstructure of emulsion gels, thereby affecting the water- and oil-holding capacity and textural properties of emulsion gels. Similarly, Li et al. [38] reported that appropriate addition of *Konjac glucomannan* improved the emulsification properties of MP and contributed to the formation of network structure. As shown in Figure 5 and Figure 6, the observed trend in the microstructural changes in Cur/MP emulsion gels was consistent with Figure 2 WHC and Figure 3 rheological behavior results. This reinforced gel network structure contributed to enhanced WHC and exhibited favorable gel elasticity.

### 2.3. Emulsified Surimi Gels Property Determination

#### 2.3.1. TPA and Gel Strength

TPA and gel strength are critical indicators for assessing surimi gel quality. As shown in Table 3, pure MP emulsion gel did not significantly improve the gel strength or TPA properties of surimi gels compared to those with added soybean oil (*p* > 0.05). Pei et al. [12] similarly observed that incorporating pure tilapia MP emulsion gel weakened surimi gel strength due to the aqueous phase in the MP emulsion gel. In contrast, adding Cur/MP emulsion gel significantly enhanced gel strength, hardness, chewiness, and gumminess of surimi gels at 4–6% Cur concentrations (*p* < 0.05), with optimal values at 6% Cur. Numerous studies have shown that pre-emulsification reduces the particle size of oil droplets and thus decreases their damage to the protein gel network [27]. Figure 5 and Figure 6 demonstrated that increasing Cur concentration reduced oil droplet size and improved uniformity in Cur/MP emulsion gels. Additionally, the texture of the surimi gel was hardened due to the thickening and gelling properties of Cur.

#### 2.3.2. WHC and Whiteness

Surimi gels form elastic colloids by trapping water through a protein network. Compact and homogeneous gel network exhibits excellent WHC [39]. As shown in Table 3, emulsion gel addition significantly improved surimi gel WHC compared to soybean oil incorporation (*p* < 0.05). At 6% Cur concentration, WHC increased by 9.41% relative to the control group. This enhancement can be attributed to the smaller oil droplets in the emulsion gel minimizing the interference of cross-linking between surimi proteins [27].

As shown in Table 3, whiteness was significantly higher in the control group than in the emulsion gel group (*p* < 0.05). This difference was attributed to soybean oil enhancing light scattering in surimi gels [40]. This may also be attributed to fading caused by increased light scattering from smaller droplets, as light waves penetrate less deeply into the emulsion before being backscattered, resulting in reduced light absorption [41]. Furthermore, whiteness increased with higher Cur concentrations in emulsion gel formulations. This may stem from improved light scattering properties due to structural modifications induced by Cur/MP emulsion gels in the surimi matrix [32]. The exceptional ability of Cur to bind water molecules and the denser three-dimensional gel network structure induced by Cur addition both contributed to the retention of more water and oil droplets, which ultimately enhanced light scattering [11].

#### 2.3.3. LF-NMR

**Figure 7:**
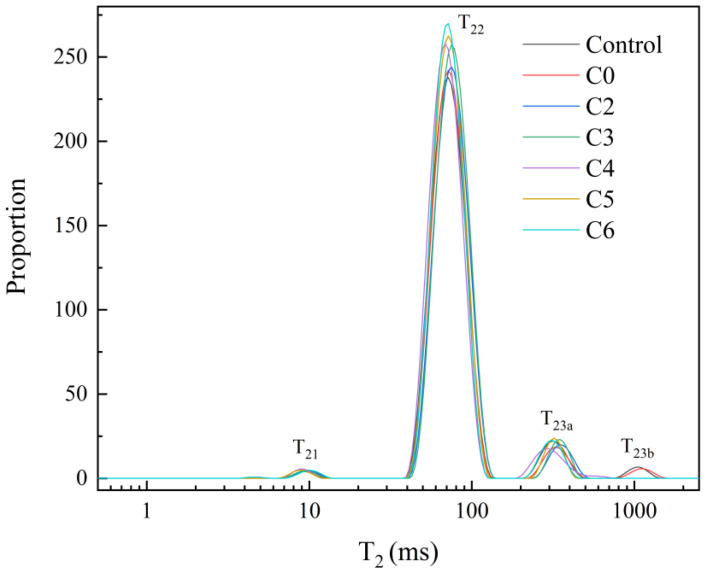
Spin-spin relaxation spectra (T_2_) of emulsified surimi gels.

Table 4 and Figure 7 show the effect of soybean oil and Cur/MP emulsion gel with varying Cur contents on the water distribution of surimi gels. Compared with the control group, the addition of emulsion gel promoted the migration of free water to immobilized and bound water (*p* < 0.05), which was consistent with the results of WHC. This suggested that pre-emulsification facilitates the reduction in network structure disruption by oil droplets. As shown in Table 4, Cur/MP emulsion gel with high Cur content (4–6%) significantly reduced the water mobility in surimi gels, with C6 group exhibiting the highest proportion of immobilized and bound water (*p* < 0.05). These findings collectively demonstrate that Cur/MP emulsion gels enhanced structural integrity in emulsified surimi gels.

## 3. Conclusions

In this research, the Cur/MP emulsion gels were prepared by adding different proportions of Cur, and the feasibility of improving the gel properties of MP emulsion gels by Cur was confirmed. The results indicated that Cur had a filling role and enhanced the strength of the network structure in Cur/MP emulsion gels. The high concentration (6%) of Cur dominated the continuous phase of the emulsion gel, resulting in a substantial enhancement of the hardness of the MP emulsion gel. Cur formed a composite network structure with MP molecules mainly through hydrogen bonding interactions, thus promoting the migration of free water to immobilized water in the emulsification system, ultimately exhibiting better gel elasticity. Moreover, the addition of Cur enhanced the adsorption of MP at oil/water interface and improved the emulsion stability. Compared with the pure MP emulsion gel, the Cur/MP emulsion gel had superior water-holding properties, gel texture, rheological properties and microstructure. In conclusion, this study presents a simple and efficient method for the construction of Cur/MP emulsion gels and elucidates the effect of Cur on the performance of Cur/MP emulsion gels. This study provides a reference for improving the utilization of low-value fish protein and meeting public demand for dietary health. Future research should further investigate the mechanism by which the Cur/MP emulsion gel improves the gel properties of emulsified surimi gels and evaluate the effects of its addition on the sensory properties and shelf life of surimi products.

## 4. Material and Methods

### 4.1. Materials and Chemicals

Frozen surimi of *Nemipterus virgatus* used in this study was purchased from Qingdao Tengbenwei Foods Co., Ltd. (Qingdao, China). Cur (food grade), supplied by Jiangsu Yiming Technology Co., Ltd. (Taixing, China), consists of molecules with 300–500 glucose residues, an average polymerization degree of 450, and a molecular weight of approximately 74,000. Soybean oil was procured from Yonghui Fresh Food Supermarket (Guangzhou, China). Nile Red and Nile Blue were purchased from Shanghai Yuanye Biotechnology Co., Ltd. (Shanghai, China). Calcofluor White was available from Sigma-Aldrich Co., Ltd. (Shanghai, China). All other chemicals were of analytical grade and purchased from Sinopharm Chemical Reagent Co., Ltd. (Shanghai, China).

### 4.2. Extraction of MP

MP was extracted from frozen surimi of *Nemipterus virgatus* following the method of Lv et al. [42]. The extraction process was maintained at a low temperature of 4 °C throughout, and the concentration of MP was determined using the Biuret method [43].

### 4.3. Preparation of Cur/MP Mixtures

The MP concentration was diluted to 20 mg/mL with phosphate-buffer solution (0.6 M NaCI, pH 7.4). Subsequently, different proportions of Cur powder were added to the MP suspension and continuously stirred for 2 h to form a mixed homogeneous dispersion. The concentrations of Cur added into MP suspension were 0, 2, 3, 4, 5 and 6% (*w*/*v*), respectively. After being heated at 40 °C for 20 min, the samples were immediately transferred to a water bath preheated to 90 °C and held for 20 min. Following heating, the gels were rapidly cooled in crushed ice for 1 h. Finally, they were stored in a 4 °C refrigerator until further testing.

### 4.4. Characterization of Cur/MP Mixtures

#### 4.4.1. Fourier Transform Infrared Spectrometer (FTIR)

The Cur/MP mixture was lyophilized for 48 h and homogenized into a powder for testing. The powder was mixed with KBr at a 1.5:100 (*w*/*w*) ratio, ground and pressed into a thin sheet. Subsequently, spectra were collected in the 4000–400 cm^−1^ range in 64 scans by a Vertex-70 spectrophotometer (Bruker Technologies Ltd., Billerica, MA, USA).

#### 4.4.2. X-Ray Diffraction (XRD)

X-ray diffraction analyses of pure Cur, pure MP, and Cur/MP mixtures with varying Cur concentrations were carried out using an X-ray diffractometer (Ultima IV; Kuraray Co., Ltd., Tokyo, Japan) in reflection mode at 40 kV and 40 mA. The 2*θ* range was scanned from 10° to 60° with a step size of 0.02° [27].

### 4.5. Preparation of Cur/MP Emulsion Gels

Cur powder was added to MP suspensions at designated ratios then uniformly dispersed (as described in Section 2.3). The Cur/MP suspension and soybean oil were emulsified at a high speed of 12,000 rpm for 2 min to form emulsified systems, and the volume of the oil phase *φ* = 50%. Subsequently, emulsions were incubated at 40 °C for 30 min and then heated to 90 °C for 20 min to obtain Cur/MP emulsion gels, rapidly cooled, and stored overnight at 4 °C for subsequent characterization.

### 4.6. Cur/MP Emulsion Gels Property Determination

#### 4.6.1. Texture Profile Analysis (TPA) and Gel Strength

TPA and gel strength of the samples were analyzed by TA-XT texture analyzer (TA-XT plus, Stable Micro Systems Ltd., Godalming, UK). After equilibrating to room temperature, the emulsion gel samples were cut into cylinders with a height of 20 mm for subsequent analysis. TPA was performed according to the method described by Yang et al. [44] with slight modifications. The TA/50 probe was used under the following parameters: constant test speed of 1 mm/s, trigger force of 5 g, and engineering strain of 50%. Gel strength was determined based on the method of Jiang et al. [17] with minor adjustments. Measurements were carried out using a TA/0.5 probe, which penetrated the gel axially to a depth of 15 mm at a speed of 1.5 mm/s. All measurements were performed in triplicate.

#### 4.6.2. Water-Holding Capacity (WHC)

The WHC of the sample was measured by referring to the method of Lv et al. [45] with minor modifications. Briefly, emulsion gel samples (5 g) were centrifuged for 10 min (10,000× *g*, 4 °C). Surface moisture was removed using filter paper. The WHC was calculated as the percentage ratio of post-centrifugation to pre-centrifugation sample weight.

#### 4.6.3. Rheological Properties

The rheological properties of emulsion gels were analyzed using a rheometer (HR20, TA Corporation, Santa Fe Springs, CA, USA) equipped with T20 parallel plates at a gap distance of 1 mm with reference to previous methods [34]. The parameters of the strain scan are shown below: frequency 1 Hz, shear strain range 0.01–100%, temperature 25 °C. A strain of 0.1–1% in the viscoelastic region (LVE) was determined from the strain scan. The parameters for the frequency scan were as follows: shear strain 1%, frequency 0.1–10 Hz, temperature 25 °C.

#### 4.6.4. Low-Field Nuclear Magnetic Resonance (LF-NMR)

In order to determine the effect of Cur on the stability of MP emulsion gels, the water and oil distributions of the emulsion gel samples were assessed by a nuclear magnetic resonance (NMR) analyzer (Niumag Co., Ltd., Shanghai, China). The setup parameters were consistent with those of Zhu et al. [20].

#### 4.6.5. Confocal Laser Scanning Microscopy (CLSM)

Samples were sectioned stained and imaged using a CLSM (TCS SP8 STED 3×, Leica Microsystems Inc., Wetzlar, Germany) at 20× magnification. The emulsion gel samples were stained after cutting into thin slices. Oil, protein and polysaccharide were labeled with Nile Red (1 mg/mL, excitation wavelength 488 nm), Nile Blue (1 mg/mL, excitation wavelength 633 nm) and Calcium Fluorescent White stain (1 mg/mL, excitation wavelength 405 nm), respectively.

#### 4.6.6. Cryo-Scanning Electron Microscope (Cryo-SEM)

Referring to the previous method [46], the emulsion gel samples were sublimated with liquid nitrogen at −80 °C for 15 min and sputter plated with gold. The microstructures of the emulsion gel samples were examined using a scanning electron microscope (S4800, Hitachi, Ltd., Tokyo, Japan) equipped with a Cryo-SEM preparation system (PP3010T, Quorum Technologies Ltd., Lewes, UK) at 700× magnification.

### 4.7. Preparation of Emulsified Surimi Gels

Frozen surimi thawed overnight at 4 °C, was mixed with 2.5% NaCl for 1 min. Subsequently, 10% unheated Cur/MP emulsion gel (prepared as described in Section 4.5) with varying Cur contents was added to the surimi. These mixtures were designated C0, C2, C3, C4, C5, and C6, respectively. The control consisted of surimi supplemented with 10% soybean oil. The moisture content of all surimi mixtures was adjusted to 78% using crushed ice, followed by continuous chopping for 4 min. The surimi mixtures were then stuffed into polyvinylidene chloride casings (inner diameter 30 mm), incubated at 40 °C for 30 min followed by 90 °C for 20 min, rapidly cooled. After heat treatment, all samples were stored overnight at 4 °C.

### 4.8. Emulsified Surimi Gels Property Determination

#### 4.8.1. TPA and Gel Strength

TPA and gel strength of the samples were analyzed using a TA-XT plus texture analyzer (Stable Micro Systems Ltd., Godalming, UK). After equilibrating to room temperature, the surimi gel samples were cut into cylinders with a height of 20 mm for subsequent analysis. The TPA and gel strength of the surimi gel samples were measured using the same test parameters as those described in Section 4.6.1. Each sample was analyzed with three replicates.

#### 4.8.2. WHC and Whiteness

WHC and whiteness were measured according to the previous method [8] with slight modifications. For WHC determination, a surimi gel sample (3 g) was wrapped in filter paper and centrifuged at 10,000× *g* for 10 min at 4 °C. WHC was calculated as the percentage of the sample weight retained after centrifugation relative to its initial weight.

A colorimeter (CR-410, Konica Minolta Camera, Co, Tokyo, Japan) was used to determine the lightness (L*), red-green value (a*), and yellow-bulb value (b*) of surimi gels with a height of 20 mm. All measurements were performed in triplicate. Whiteness was calculated as follows:

Whiteness = 100 − [(100 − L*)^2^ + a*^2^ + b*^2^]^1/2^

#### 4.8.3. LF-NMR

The moisture distribution of surimi gel was determined according to the method of Zhou et al. [47] using low-field NMR (MesoMR23-040V-I; Niumag Analytical Instrument Corporation, Shanghai, China). Cylindrical surimi gel samples (30 mm thickness) were prepared and placed in NMR tubes. Spin-spin relaxation time (T_2_) was measured at 32 °C. Each sample was analyzed with three replicates.

### 4.9. Statistical Analysis

All experiments were repeated three times and data were expressed as mean ± standard deviation. Significance analysis was performed using SPSS 22.0 software (IBM Corp., Armonk, NY, USA), with and one-way analysis of variance (ANOVA) applied to determine the data for significant differences. Significantly different groups (*p* < 0.05) were denoted by distinct superscript letters. Tukey’s Honestly Significant Difference test was performed on all data following analysis of ANOVA.

## Figures and Tables

**Table 1:** Effects of different Cur concentrations on gel strength and TPA of Cur/MP emulsion gels.

Concentration of Cur	Gel Strength (g·mm)	Hardness (g)	Springiness (cm)	Gumminess (g)	Chewiness (mJ)	Cohesiveness
Control	105.85 ± 6.34 ^e^	125.06 ± 5.78 ^f^	7.60 ± 0.02 ^c^	75.24 ± 10.25 ^e^	57.29 ± 9.28 ^e^	0.6 ± 0.06 ^c^
2%	241.06 ± 20.08 ^d^	247.82 ± 18.77 ^e^	8.06 ± 0.00 ^b^	136.73 ± 21.44 ^d^	110.12 ± 16.96 ^d^	0.55 ± 0.06 ^cd^
3%	316.36 ± 23.73 ^c^	306.39 ± 24.46 ^d^	7.96 ± 0.02 ^b^	158.20 ± 11.6 ^d^	125.86 ± 7.18 ^d^	0.52 ± 0.00 ^e^
4%	378.28 ± 65.87 ^c^	424.04 ± 36.6 ^c^	8.76 ± 0.02 ^a^	290.45 ± 22.33 ^c^	254.59 ± 20.58 ^c^	0.69 ± 0.04 ^b^
5%	481.04 ± 72.29 ^b^	521.05 ± 5.76 ^b^	8.65 ± 0.01 ^a^	384.47 ± 9.19 ^b^	332.52 ± 14.26 ^b^	0.74 ± 0.02 ^ab^
6%	734.89 ± 64.97 ^a^	736.45 ± 30.76 ^a^	8.66 ± 0.05 ^a^	568.19 ± 21.57 ^a^	492.06 ± 21.41 ^a^	0.77 ± 0.00 ^a^

Different letters within the same column indicate statistically significant differences (*p* < 0.05).

**Table 2:** Effects of different Cur concentrations on T_2_ relaxation peak relative area of Cur/MP emulsion gels.

Concentration of Cur	T_2_ Relaxation Peak Relative Area (%)		
	A_21_	A_22_	A_23_
Control	0.03 ± 0.02 ^b^	79.87 ± 0.71 ^d^	20.10 ± 0.68 ^a^
2%	0.04 ± 0.01 ^ab^	84.11 ± 1.99 ^c^	14.64 ± 1.04 ^b^
3%	0.03 ± 0.01 ^b^	86.83 ± 1.41 ^bc^	13.14 ± 1.41 ^b^
4%	0.05 ± 0.02 ^ab^	87.67 ± 3.31 ^b^	12.28 ± 3.3 ^b^
5%	0.07 ± 0.02 ^a^	99.93 ± 0.02 ^a^	-
6%	0.07 ± 0.03 ^a^	99.93 ± 0.03 ^a^	-

Different letters within the same column indicate statistically significant differences (*p* < 0.05).

**Table 3:** Gel strength, TPA, WHC and whiteness of emulsified surimi gels.

Sample Name	Gel Strength (g·mm)	Hardness (g)	Springiness (cm)	Gumminess (g)	Chewiness (mJ)	Cohesiveness	WHC	Whiteness
Control	2276.94 ± 121.62 ^d^	3363.11 ± 45.82 ^d^	8.99 ± 0.00 ^a^	2778.56 ± 5.21 ^d^	2498.68 ± 8.19 ^d^	0.83 ± 0.01 ^a^	82.39 ± 1.85 ^f^	81.45 ± 0.31 ^a^
C0	2327.28 ± 141.69 ^d^	3439.76 ± 41.41 ^d^	9.08 ± 0.01 ^a^	2864.74 ± 29.92 ^d^	2601.66 ± 35.69 ^cd^	0.83 ± 0.00 ^a^	84.40 ± 0.47 ^e^	76.85 ± 0.31 ^e^
C2	2354.61 ± 107.67 ^d^	3608.62 ± 64.26 ^c^	9.03 ± 0.03 ^a^	3012.90 ± 23.68 ^d^	2720.11 ± 106.41 ^bc^	0.84 ± 0.01 ^a^	85.20 ± 0.33 ^de^	77.76 ± 0.63 ^d^
C3	2470.32 ± 125.07 ^cd^	3775.74 ± 14.28 ^b^	9.05 ± 0.01 ^a^	3137.31 ± 53.47 ^d^	2837.85 ± 77.31 ^b^	0.83 ± 0.01 ^a^	86.28 ± 0.41 ^cd^	78.36 ± 0.11 ^d^
C4	2654.41 ± 107.10 ^bc^	3837.46 ± 30.98 ^b^	9.11 ± 0.01 ^a^	3149.79 ± 60.39 ^c^	2867.74 ± 39.98 ^b^	0.82 ± 0.02 ^a^	87.22 ± 0.55 ^bc^	78.46 ± 0.44 ^d^
C5	2766.86 ± 111.26 ^ab^	4111.54 ± 106.42 ^a^	9.14 ± 0.00 ^a^	3455.46 ± 152.53 ^b^	3157.24 ± 151.49 ^a^	0.84 ± 0.02 ^a^	88.31 ± 0.45 ^b^	79.35 ± 0.31 ^c^
C6	2916.58 ± 56.45 ^a^	4163.11 ± 89.49 ^a^	9.18 ± 0.02 ^a^	3481.12 ± 116.91 ^a^	3196.54 ± 133.86 ^a^	0.84 ± 0.01 ^a^	90.14 ± 0.40 ^a^	80.10 ± 0.17 ^b^

Different letters within the same column indicate statistically significant differences (*p* < 0.05).

**Table 4:** T_2_ relaxation peak relative area of emulsified surimi gels.

Samples	T_2_ Relaxation Peak Relative Area (%)			
	A_21_	A_22_	A_23_	
			A_23a_	A_23b_
Control	0.08 ± 0.01 ^c^	65.34 ± 1.50 ^d^	18.82 ± 1.28 ^b^	15.77 ± 2.56
C0	0.07 ± 0.01 ^c^	67.34 ± 0.22 ^c^	16.71 ± 0.34 ^c^	15.87 ± 0.40
C2	0.09 ± 0.01 ^bc^	77.78 ± 0.49 ^b^	22.13 ± 0.50 ^a^	-
C3	0.09 ± 0.01 ^bc^	79.57 ± 1.49 ^ab^	20.34 ± 1.50 ^ab^	-
C4	0.11 ± 0.02 ^ab^	80.08 ± 0.99 ^a^	19.80 ± 0.99 ^b^	-
C5	0.11 ± 0.02 ^a^	81.00 ± 0.64 ^a^	18.89 ± 0.63 ^b^	-
C6	0.13 ± 0.01 ^a^	80.99 ± 0.57 ^a^	18.88 ± 0.58 ^b^	-

Different letters within the same column indicate statistically significant differences (*p* < 0.05).

## Data Availability

The original contributions presented in this study are included in the article. Further inquiries can be directed to the corresponding author.
